# Altered membrane properties but unchanged intrinsic excitability and spontaneous postsynaptic currents in an aged APP_*swe*_/PS1dE9 model of Alzheimer’s disease

**DOI:** 10.3389/fncel.2022.958876

**Published:** 2022-08-26

**Authors:** Shane M. Ohline, Xinhuai Liu, Mohamed F. Ibrahim, Bruce M. Mockett, Ruth M. Empson, Wickliffe C. Abraham, Karl J. Iremonger, Peter P. Jones

**Affiliations:** ^1^Department of Physiology, School of Biomedical Sciences, University of Otago, Dunedin, New Zealand; ^2^HeartOtago, University of Otago, Dunedin, New Zealand; ^3^Brain Health Research Centre, University of Otago, Dunedin, New Zealand; ^4^Nuffield Department of Clinical Neurosciences, Oxford University, Oxford, United Kingdom; ^5^Department of Psychology, University of Otago, Dunedin, New Zealand

**Keywords:** Alzheimer’s disease, intrinsic excitability, aging, APP/PS1 double transgenic AD mouse, postsynaptic currents

## Abstract

Neuronal hyperexcitability in Alzheimer’s disease (AD) models is thought to either contribute to the formation of amyloid beta plaques or result from their formation. Neuronal hyperexcitability has been shown in the cerebral cortex of the widely used young APPswe/PS1dE9 mice, which have accelerated plaque formation. However, it is currently unclear if hyperexcitability also occurs in CA1 hippocampal neurons of aged animals in this model. In the present work, we have compared intrinsic excitability and spontaneous synaptic inputs from CA1 pyramidal cells of 8-month-old APPswe/PS1dE9 and wildtype control mice. We find no change in intrinsic excitability or spontaneous postsynaptic currents (PSCs) between groups. We did, however, find a reduced input resistance and an increase in hyperpolarization-activated sag current. These results are consistent with findings from other aged AD model mice, including the widely used 5xFAD and 3xTg. Together these results suggest that neuronal hyperexcitability is not a consistent feature of all AD mouse models, particularly at advanced ages.

## Introduction

Alzheimer’s disease (AD) pathology is characterized predominantly by the appearance of amyloid beta plaques and tau tangles. Neuronal hyperexcitability has been shown to be either a downstream effect or a potential cause of this pathology (Muller et al., 2021). One of the first papers to report hyperexcitability in a mouse model of AD (APPswe/PS1dE9) (in neocortical layer 2/3 pyramidal cells) also reported seizures in young (3–4.5-month old) animals (Minkeviciene et al., 2009). While this work represents an important snapshot of this animal model at a young age, we do not have a complete picture of excitability in older animals when the amyloid plaque burden is higher.

During subsequent years, several groups have investigated the intrinsic excitability of other mouse models of AD as well as tissue from rodent brains treated with amyloid beta peptides (Brown et al., 2011; Kerrigan et al., 2014; Siskova et al., 2014; Tamagnini et al., 2015a,b; Musial et al., 2018; Yao et al., 2020, 2022; Liu et al., 2021; Russo et al., 2021; Vitale et al., 2021). The general consensus has been that there is altered excitability in each of these models, but it varies widely from substantially hyperexcitable (Yao et al., 2020, 2022; Liu et al., 2021) to virtually no change (Musial et al., 2018; Russo et al., 2021). In Musial et al. (2018) and Russo et al. (2021), animals were investigated across age ranges from 1 to 24 months and found varying differences across ages and models. Musial et al. (2018) found dorsal CA1 neurons had exacerbated spike frequency accommodation, and this worsened with age in the three different AD models (J20, 3xTg, and 5xFAD). This was indicative of reduced excitability as the animals aged to 24 months. In the ventral CA1 Russo et al. (2021) found no changes in excitability in aged AD models (5xFAD or 3xTg) models compared to age-matched wild-type animals. Interestingly, when hyperexcitability is present, it may not be present in all neurons. Specifically, Kuchibhotla et al. (2008) showed using calcium imaging that only around 20% of cortical cells were hyperexcitable in the Tg-2576 model. Some have highlighted that normal aging and AD are similar in terms of neuronal degradation and that AD is accompanied by changes also seen in normal aging (Jones et al., 2011; Neill, 2012).

Given the apparent differences in excitability across AD models and ages this study aimed to characterize the aged APPswe/PS1dE9 model. This is of particular importance as this AD model is often used at an older age and the literature for other AD models suggests that changes in excitability cannot simply be assumed to remain consistent throughout the progression of AD. To determine how the excitability of dorsal CA1 neurons changes in older APPswe/PS1dE9 animals we performed whole-cell patch-clamp electrophysiology on CA1 neurons when the animals were approximately 8 months of age, a point when amyloid beta plaques are abundant in the hippocampus. Our results reveal that CA1 cells in aged APPswe/PS1dE9 mice show altered membrane properties as indicated by a lower input resistance and increased hyperpolarization-activated sag current. However, there was no change in rheobase, resting membrane potential, and the frequency–current curve. There was also no difference in the medium and slow-afterhyperpolarization (m- and sAHP) between APPswe/PS1dE9 and wild-type control groups. Finally, we investigated the spontaneous postsynaptic currents (PSCs) in these animals and saw no changes in either the amplitude or instantaneous frequency of events between genotypes. The APPswe/PS1dE9 model is one of the dominant models used to study AD. We show that the widely accepted view that these animals have hyperexcitable CA1 cells must be reassessed at older ages.

## Materials and methods

### Animals

All animal-use procedures were approved by the University of Otago Animal Ethics Committee (#AUP 20-91) and conducted in accordance with New Zealand Animal Welfare legislation. 8-month old (± 1 month) male and female APPswe/PS1dE9 and wild-type C57BL/6J mice [5 female (2 WT, 3 Tg), 27 male (16 WT, 11 Tg)] from the same litters were used. The APPswe/PS1dE9 mice on a C57BL/6J-congenic background harbored mutations in human APP_695_ (the Swedish mutations: K670N, M671L) and human PS1 exon nine deletion (PS1_ΔE9_). Genotyping was completed from ear notches by Transnetyx (United States). Mice were housed in groups (up to 5 same-sex mice per cage) and maintained on a 12 h light/dark cycle (lights on 6 a.m.). All animals had access to food and water *ad libitum*.

### Electrophysiology

#### Slice preparation

Eight-month-old APPswe/PS1dE9 and wild-type mice were anesthetized with pentobarbital and then transcardially perfused with ice-cold *N*-methyl-D-glucamine (NMDG) solution (in mM: 92 NMDG; 2.5 KCl; 1.2 NaH_2_PO_4_; 30 NaHCO_3_; 20 HEPES; 25 glucose; 5 sodium ascorbate; 2 thiourea; 3 sodium pyruvate; 10 MgSO_4_.7H_2_O; 0.5 CaCl_2_. 2H_2_O with pH adjusted to 7.3–7.4 with HCl) (Ting et al., 2018). This was equilibrated with 95% O_2_ and 5% CO_2_. The brain was removed from the skull and was kept in ice-cold NMDG solution while slices were prepared. Coronal sections (300 μm thick) taken from the septal pole containing both hippocampi were cut with a vibratome (Leica VT1000s). The two hemispheres were then separated and dorsal hippocampal slices were transferred to a chamber containing aCSF (in mM): 126 NaCl; 2.4 KCl; 1.5 NaH_2_PO_4_; 10 glucose; 26 NaHCO_3_; 2.4 CaCl_2_; 1.3 MgCl_2_. Slices were bubbled with 95% O_2/_5% CO_2_ and maintained at 34^°^C for 30 min and then at room temperature (23 ± 3^°^C). To conduct the whole-cell patch-clamp experiments, the slices were transferred to a submerged recording chamber where they were perfused with aCSF at 2.1 mL/min and maintained at 25 ± 1^°^C.

#### Recordings

Whole-cell patch-clamp recordings of CA1 neurons were undertaken using a fixed-stage upright microscope (Eclipse FN1; Nikon, Tokyo, Japan) under Nomarski differential interference contrast optics (60X water-immersion objective). Recordings were performed using microelectrodes (4–6M Ω, borosilicate thin walled with filament, Warner Instruments G150TF-4, pulled using a Sutter P-97 micropipette puller) filled with (in mM): 130 potassium gluconate; 10 HEPES; 4 sodium ATP; 0.4 sodium GTP; 10 phosphocreatine; 4 MgCl_2_; 6.2 neurobiotin (Vector labs, SP-1120) with the pH adjusted to 7.35 with KOH and the osmolality to 295 mOsm.

Signals (voltage and current) were amplified with a Multiclamp 700 B amplifier (Molecular Devices, Foster City, CA) and digitized with a Digidata 1440A (Molecular Devices). Signals were filtered with the Bessel filter of Multiclamp 700B (at 3 kHz for current or 10 kHz for voltage) before being digitized at 20 kHz. Acquisition and subsequent analysis of the acquired data were performed with the Clampex 10 suite of software (Molecular Devices), Easy Electrophysiology 2.4.0 (Oxford), and GraphPad Prism 9.4.1 (GraphPad Software, San Diego, United States). Resting membrane potential was determined in the current clamp without any holding current and liquid junction potentials of ∼ 12 mV for the gluconate-based solution were not corrected.

#### Analysis

Criteria to include cells in the analysis were an absolute leak current < 100 pA at holding potential (−70 mV), and an access resistance of < 25 MΩ. The membrane resistance and capacitance were monitored, although no criteria were set for these measures for inclusion. Input resistance was assessed by the application of current steps of 25 pA in current-clamp configuration after taking the membrane potential to −70 mV. Seven current steps from −100 pA to + 50 pA were used to plot a V-I curve, the slope of which was taken as the input resistance (see Supplemental Figure 1 for representative plots). All excitability measurements were obtained from positive current steps up to + 375 pA. The membrane potential threshold to fire the first action potential was measured by taking the first derivative of the first action potential. The threshold was identified to be the point where the magnitude was 5% of the maximum of the first derivative. The action potential height was taken from the threshold to the maximum height of the first action potential. The action potential width was measured in Clampfit 10.7 as the width at half-maximum height.

**FIGURE 1:**
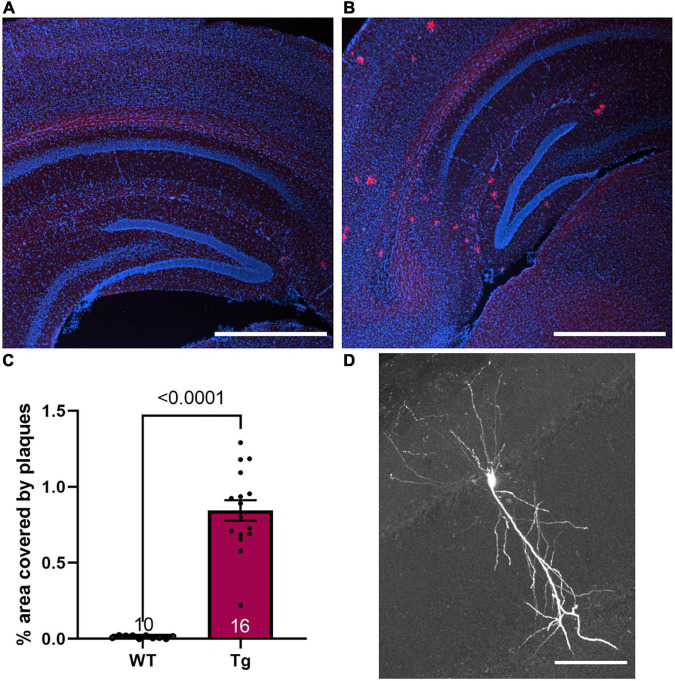
Plaque phenotype is present in 8-month-old Tg mice. **(A,B)** Fluorescence image of nuclei (DAPI, blue) and Congo red stain (red) merged in **(A)** a WT mouse and **(B)** a Tg mouse. Scale bar 1 mm. **(C)** The average plaque area in the hippocampus was 0.85 ± 0.07% in 8-month-old Tg mice (*n* = 16) while the background fluorescence in the WT animals results in an area of 0.01 ± 0.003% (*n* = 10). **(D)** A representative CA1 neuron filled with biotin during whole-cell patch clamp recordings. Scale bar 100 μm.

The slow and medium AHP were obtained by holding the cell at −60 mV, injecting 4 × 2 nA current pulses (2 ms long) separated by 70 ms (start to start) to elicit four action potentials, and maintaining the recording for a total of 2 s. The measurements are outlined in Figure 4A. To estimate hyperpolarization-activated cyclic nucleotide-gated (HCN) channel activity, we measured both the “sag” and the rebound voltages from the first four hyperpolarizing current steps (−100 to −25 pA). We measured the SAG ratio as


$$S\,A\,G\,r\,a\,t\,i\,o=\frac{V_{S\,A\,G}+\left(V_{S\,S}-V_{h}\right)}{\left(V_{S\,S}-V_{h}\right)}$$

**FIGURE 2:**
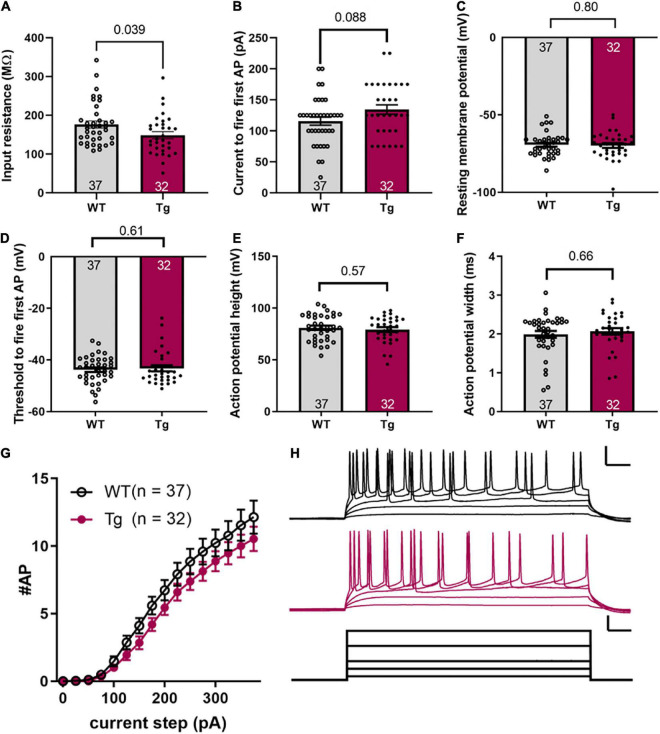
Intrinsic excitability is not different between WT and Tg mice. **(A)** Input resistance, **(B)** current to fire the first action potential, **(C)** resting membrane potential, **(D)** threshold voltage to fire first action potential, **(E)** action potential height, **(F)** action potential width (FWHM), **(G)** number of action potentials fired for a given current step. **(H)** Representative data for a single WT and Tg mouse. Upper scale bars, 50 ms and 25 mV. Current scale bars, 50 ms and 100 pA. Error bars indicate ± SEM; the number of cells (N) is indicated at the bottom of each histogram; and, the *p* value is noted above the connecting line at the top (Student’s *t*-test or Mann–Whitney as mentioned in the text).

**FIGURE 3:**
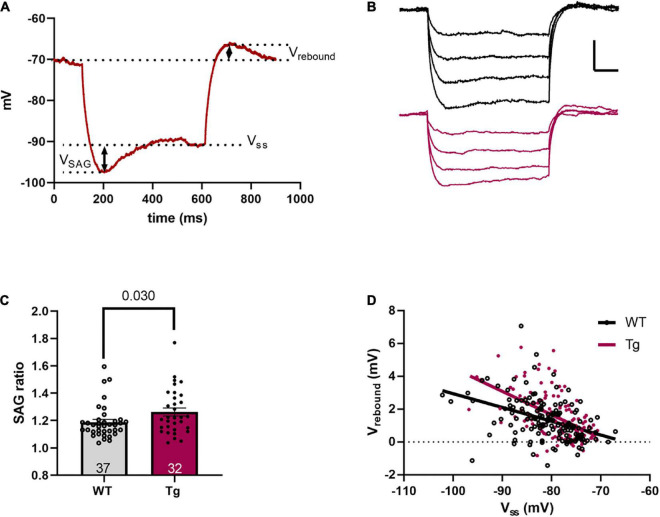
Sag and rebound are increased in 8-month-old Tg mice. **(A)** A representative voltage response to a –100 pA hyperpolarizing current step. Measurements of sag voltage (V_*SAG*_), steady-state voltage (V_*SS*_), and rebound voltage (V_*rebound*_) are noted. **(B)** Representative voltage traces resulting from four hyperpolarizing current steps (−100 to −25 pA). The black trace is a WT and the red trace is a Tg cell. Scale bar is 5 mV/100 ms. **(C)** The SAG ratio, as defined in the text, is higher in the Tg animals. **(D)** A plot of V_*rebound*_ vs. V_*SS*_ shows a significant difference in slope, indicating a larger slope in the Tg animals.

**FIGURE 4:**
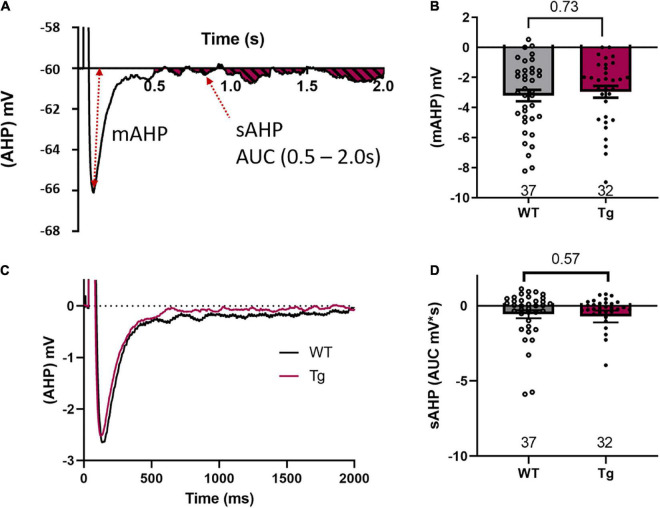
mAHP or sAHP are not different between genotypes. **(A)** A representative trace to indicate the measurement of the medium after-hyperpolarization (mAHP) and the area under the curve (AUC) measurement between 500 and 2000 ms of the slow after-hyperpolarization (sAHP). **(B)** mAHP results show no difference between the WT and the Tg animals. **(C)** A grand average of all WT and Tg AHP experiments indicates no difference in either the mAHP or the sAHP between the two genotypes. **(D)** The AUC measurements of the sAHP are not different between the two genotypes.

where the holding potential (V_*h*_), steady state voltage (V_*ss*_), sag voltage (V_*SAG*_), and rebound voltage (V_*rebound*_) are measured as in Figure 3A.

After attaining a stable whole-cell voltage-clamp recording held at −70 mV, spontaneous PSCs were recorded for 300 s. One to four cells were obtained from each animal. These cells were from the same animals as the excitability study. Thirteen Tg (2 F/11 M) and 13 WT animals (2F/11 M) were used for these experiments. A cell was considered acceptable if its access resistance (R_*a*_) ≤ 25 MΩ and changed by < 20% during the data collection. PSCs were detected by the MiniAnalysis program (Synptosoft Inc., NJ, United States) with a threshold of 9 pA. Each event was confirmed by visual inspection. Analysis parameters included instantaneous frequency (1/inter-event interval in s) and amplitude.

### Plaque measurements

#### Congo red staining for plaques

Following slicing for the electrophysiology as above, the remaining portion of the brain containing the ventral hippocampus was post-fixed for 24 h in 4% paraformaldehyde. This brain tissue was then placed in 30% sucrose in 0.1 M phosphate-buffered saline at 4^°^C until subsequent slicing. Slices (40 μm) were obtained from the ventral hippocampus. Five slices per brain were placed on slides. Congo red was used to stain the sections to reveal amyloid plaques (in the APPswe/PS1dE9 mice) with nuclei counter-stained with DAPI. Congo red staining and DAPI were visualized on a Nikon Eclipse Ti2 fluorescence microscope. Images of Congo red and DAPI were captured using a Nikon DC Qi2 camera, a 10x objective (Plan Apo; N/A = 0.30; Nikon, Tokyo, Japan) and NIS-Element F 4.6 software. Images were converted to 8-bit, a threshold value was determined and maintained for all images, and the percentage area covered in the whole hippocampus by plaques was calculated using ImageJ.

### Statistical analysis

All analyses were performed using Graphpad Prism 9.4.1.

#### Current clamp measurements

Unless otherwise specified, data are presented as mean ± SEM. If the data were normally distributed with equal variances (as determined by a Shapiro–Wilk test), a parametric Student’s *t*-test was applied. If data failed a Shapiro–Wilk test a non-parametric Mann-Whitney test was used. N denotes the number of cells, while the number of animals was 18 WT and 14 Tg. When required, either an ANOVA or an ANCOVA was performed. In all cases, significance was indicated by *p* < 0.05 with Bonferroni’s *post-hoc* tests for parametric tests.

#### Voltage clamp measurements

For PSCs, statistical analysis of the mean instantaneous frequency and amplitude was undertaken using a Mann-Whitney test. To compare cumulative frequency the Kolmogorov-Smirnov test was used. In all analyses, differences were considered statistically significant at *p* < 0.05.

## Results

For all measurements, APPswe/PS1dE9 mice are denoted as Tg with their wild-type littermates denoted as WT. To confirm the phenotype of the Tg mice, Congo red staining was performed. Sections from WT mice showed no plaques, although low-level background staining was evident at the threshold applied. Tg mice all showed plaque formation throughout the cortex and the hippocampus with the average hippocampal area covered by plaques being 0.85 ± 0.07% (Figures 1A–C). These results indicate a strong plaque phenotype was established in these 8-month-old mice.

Intrinsic excitability measurements were conducted on hippocampal CA1 pyramidal cells, identified by their morphology (see Figure 1D) and location within the brain slice. The input resistance was reduced in APPswe/PS1dE9 mice (Figure 2A, WT: 176.3 ± 9.1 MΩ; and Tg: 148.6 ± 9.1 MΩ, *p* = 0.039, Mann-Whitney test). The current step required to fire the first action potential (rheobase) was not different between the two groups (Figure 2B, WT: 115.5 ± 6.4 pA and Tg: 134.4 ± 7.6 pA, *p* = 0.088, Mann-Whitney test). The resting membrane potential was not different between genotypes (Figure 2C, WT: −69.3 ± 1.2 mV; Tg: −69.8 ± 1.6 mV; *p* = 0.80, Students *t*-test). The voltage threshold to fire the first action potential was not different between the two groups (Figure 2D, WT: −43.78 ± 0.88 mV; Tg: −43.27 ± 1.17 mV, p = 0.61, Mann-Whitney test). The height of the action potential (from threshold to peak) was not different between groups (Figure 2E, WT: 80.99 ± 2.07 mV; Tg: 79.2 ± 2.26 mV, *p* = 0.57, unpaired *t*-test), nor was the width at half-maximum (Figure 2F, WT: 1.99 ± 0.088 pA; Tg: 2.07 ± 0.084 pA, *p* = 0.66, Mann-Whitney test). The number of action potentials fired at a given current step was also not different between the two groups [Figure 2G, *p* = 0.18, *F*_(1,67)_ = 1.8 for genotype; *p* = 0.54, *F*_(15,991)_ = 0.92 genotype × current step, in a mixed-effects analysis]. Figure 2H shows representative data used to generate the results shown in Figure 2G. These results indicate that the 8-month-old APPswe/PS1dE9 mice have altered membrane resistance, but unchanged intrinsic excitability compared to the WT animals.

To investigate if the underlying difference in input resistance was likely due to altered HCN channel activity, we measured the “sag,” steady state, and rebound voltages in response to hyperpolarizing current steps. Figure 3A shows the method of measurement for sag and rebound, while Figure 3B shows representative traces for WT and Tg cells. The SAG ratio was larger in the APPswe/PS1dE9 mice (WT: 1.19 ± 0.02; and Tg: 1.26 ± 0.03; *p* = 0.03, Mann-Whitney test, Figure 3C). Plotting V_rebound_ vs. V_SS_ showed that the regression line was steeper in the APPswe/PS1dE9 mice (Figure 3D (rebound) WT: *m* = −0.084 ± 0.015; and Tg: −0.14 ± 0.020; F_1,270_ = 5.45, *p* = 0.020, two-tailed ANCOVA). These results indicate that the hyperpolarization-induced sag current was larger in the Tg animals, consistent with the reduced input resistance for these animals.

We then measured the medium after-hyperpolarization (mAHP) and found no significant difference between genotypes in mAHP amplitude (WT: −3.2 ± 0.38 mV; Tg: −2.95 ± 0.39 mV; *p* = 0.73, Mann–Whitney test, Figure 4B). We also measured the slow after-hyperpolarization (sAHP) by calculating the area under the curve (AUC) from 500 to 2000 ms after a burst of action potentials. The AUC was measured both below and above the holding voltage (−60 mV, Figure 4A). A grand average of the data from our measurements of both m- and sAHP is shown in Figure 4C. However, there was no significant difference between genotypes in the AUC for the sAHP (Figure 4D, WT: −0.57 ± 0.27 mV*s; and Tg: −0.71 ± 0.39 mV*s; Mann-Whitney test, *p* = 0.57).

**FIGURE 5:**
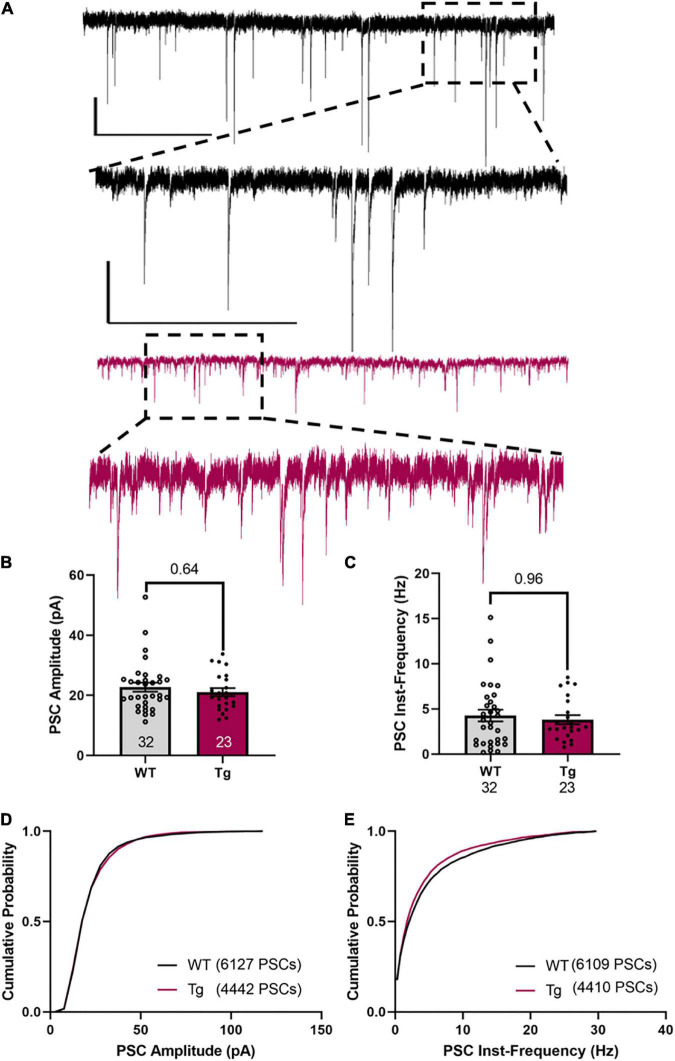
sPSCs in CA1 neurons are not different between WT and Tg mice. **(A)** Representative traces of PSCs. Upper traces (black) are from a WT animal. The lower two traces (red) are from a Tg animal. The scale bars are 20 pA/5 s in the top trace and 10 pA/500 ms in the inset trace. These are the same for both genotypes. **(B)** The average amplitude is not different between the genotypes. **(C)** The instantaneous frequency between events is not different between the genotypes. Cell N is indicated at the bottom of histograms. **(D)** A cumulative plot of the amplitude of pooled sPSCs [6141 amplitude of sPSCs from 32 cells (WT), 4450 amplitude of sPSCs from 23 cells (Tg)] showing there is no statistical difference between WT and Tg (*p* > 0.9, Kolmogorov-Smirnov Test). **(E)** A cumulative plot of the inst-frequency of pooled sPSCs [6109 inst-frequency of sPSCs from 32 cells (WT), 4427 inst-frequency of sPSCs from 23 cells (Tg)] showing there is no statistical difference between WT and Tg (*p* > 0.8, Kolmogorov–Smirnov test).

Measuring the amplitude and frequency of the spontaneous PSCs (Figure 5A), showed that the WT and Tg mice were not different (Figure 5B, average amplitude WT: 22.7 ± 1.5 pA; Tg: 21.1 ± 1.3 pA; p = 0.64, Mann–Whitney test) and (Figure 5C, average instantaneous frequency, WT: 4.3 ± 0.64 Hz; Tg: 3.8 ± 0.50 Hz; p = 0.96, Mann–Whitney test). For the cumulative population distributions, there were also no differences between the genotypes (cumulative population of amplitude, Figure 5D, p = 0.92; cumulative population of instantaneous frequency, Figure 5E, p = 0.99, Kolmogorov–Smirnov test).

## Discussion

AD is characterized by cognitive impairment, even at its earliest stages. Memory loss is the most commonly identified impairment, and autobiographical memory has been shown to be affected when CA1 is damaged in humans (Bartsch et al., 2011). The hippocampus is also one of the first regions of the brain to be affected by amyloid beta plaque deposition, both in humans and in animal models of the disease. Moreover, many interventions to restore memory function in AD models have targeted the hippocampus and assessed synaptic transmission and plasticity in area CA1 (Setti et al., 2017; Tan et al., 2018). For these reasons, we chose this area for studying changes in intrinsic excitability in APPswe/PS1dE9 mice, one of the most widely used genetic mouse models of AD.

In the APPswe/PS1dE9 model of AD, Minkeviciene et al. (2009) previously examined the intrinsic excitability of neocortical layer 2/3 pyramidal neurons in young animals (aged 3–4.5-month-old). This model, particularly the C57BL/6J background strain, shows seizures at very early ages within the age window previously examined. In fact, in our breeding colony, by 5 months old, approximately 20% of the APP/PS1 animals die, presumably following spontaneous seizures as observed on occasion. In these young AD animals, Minkeviciene et al. (2009) found a depolarized resting membrane potential, and a lower current input was required for firing but with no accompanying change in input resistance. This work was accompanied by video-electroencephalogram (EEG) recordings in which at least a single unprovoked seizure was detected in 65% of the APPswe/PS1dE9 mice. This work has so dominated the field that a recent review article has focused entirely on the hyperexcitability in this model (Muller et al., 2021).

To date, no one has examined the intrinsic excitability of CA1 cells in the APPswe/PS1dE9 model, when the plaque burden is significant. At 8 months of age, these APP/PS1 mice should more closely resemble a human AD patient with advanced plaque load. We found that the plaque burden in the mice used in this study was comparable to Tan et al. (2018) and Ohline et al. (2022) where 0.8–1.1% of the hippocampal area was covered by plaques. In these animals, we measured input resistance across both hyperpolarizing and depolarizing steps and show that it was lower in CA1 pyramidal cells and that this was likely caused by an increase in sag current. This was probably due to an increased activity of the HCN channels, but our measurements do not rule out a contribution of other channels such as K_*ir*_. Despite the changes in input resistance, no direct measures of intrinsic excitability were different. Whilst all of our recordings were performed at 25^°^C allowing a direct comparison between genotypes, our absolute values are not comparable with some other studies performed at higher temperatures in other AD animal models (32–34^°^C; Neuman et al., 2015; Musial et al., 2018; Russo et al., 2021) due to the temperature sensitivity of ion channels (Van Hook, 2020). We also found that the sPSCs were not altered in APPswe/PS1dE9 mice, consistent with our previous report of no change in evoked field excitatory postsynaptic potentials in stratum radiatum of these animals although a decrease in LTP in this pathway was exhibited (Tan et al., 2018). In agreement with our results, Neuman et al. (2015) also found that the basal synaptic function in CA1 was similar in aged (11–15 mo) 5 × ADTg and WT mice, indicating a similar lack of change in an alternate model of AD.

Kuchibhotla et al. (2008) showed by calcium imaging that APPswe/PS1dE9 mice (5–6 months old) have increased calcium overload [(Ca^2+^)_*i*_] in cortical neurites. However, this overload, suggestive of hyperexcitability, was only found in approximately 20% of neurites. Whether similar effects occur in the hippocampus is not known. Due to the nature of whole cell patch-clamp electrophysiology, a limitation of our study could be that we may have a selective bias toward healthier cells, of which only a fraction might be hyperexcitable. Although in our study we patched a relatively large number of cells, selected randomly within the CA1, we set the criterion of a leak current of < 100 pA to hold the cell at −70 mV. This may have excluded unhealthy cells with potentially hyperexcitable properties, although generally, cells with large leak currents fired far fewer action potentials. This could have contributed to the lack of significant differences between the WT and APPswe/PS1dE9 animals in terms of excitability measurements.

If we examine the work to date on the intrinsic excitability of CA1 pyramidal cells in different *aged* AD models (Brown et al., 2011; Musial et al., 2018; Russo et al., 2021) our results are not dissimilar. In general, there are very small alterations in action potential properties, AHP and sag. Each aged model shows some difference from wild-type, but the differences are slight, albeit often in the direction of hyperexcitability. In 10-month-old PSAPP (APP_*swe*_/PS1_*M*146L_) mice, Brown et al. (2011) found changes in action potential waveforms and altered Na^+^ current density. However, no change in resting membrane potential or input resistance was observed. At the onset of a weak depolarizing current pulse, more burst firing was found. Tamagnini et al. (2015a) found in 20–23-months-old PDAPP (APP_*V*717F_) mice no changes in input resistance, resting membrane potential, or sag. No differences in overall number of action potentials were found, but higher instantaneous firing frequencies after accommodation occurred. This was associated with a more pronounced AHP, an altered capacitance, and an altered action potential waveform. In another APPPS1 model (APP_*swe*_/PS1_*L*166P_) in 10-month-old animals, the I_*h*_ current associated with HCN channels was larger, alterations in the membrane time constant and the action potential width were present in the transgenic animals. This was accompanied by a weak alteration in firing behavior (Vitale et al., 2021).

Finally, in a comprehensive study of aged (from 1 to 24 months old) AD model animals, including 5xFAD and 3xTg mice, Musial et al. (2018) found, like our current results, a larger rebound slope (cf Figure 3D) in aged Tg vs. WT mice. However, they also saw a depolarized membrane potential in the Tg animals. In the same group, in the *ventral* CA1, no changes in intrinsic excitability were found in either aged 3xTg or 5xFAD mice (Russo et al., 2021).

## Conclusion

In conclusion, our results indicate that intrinsic excitability is not changed in dorsal CA1 pyramidal neurons in aged APPswe/PS1dE9 mice. The only difference observed was a lower input resistance that is likely caused by increased HCN channel activity, which would need to be confirmed in future studies. We also found that spontaneous synaptic activity in these animals is not different from WT. These findings are more in line with normal aging in rodents in which CA1 neurons become hypoexcitable and CA3 neurons become hyperexcitable (Oh et al., 2016). Thus, it is likely that the accelerated aging seen in AD could amplify this excitability pattern (Oh et al., 2016). Because plaques are still abundant in these 8-month-old animals, it is likely that any influence of plaque formation would persist. Therefore, although suggested by others (Minkeviciene et al., 2009; Busche et al., 2012), perhaps amyloid beta plaques alone are not responsible for the hyperexcitability changes in the CA1 in the young animals. This work cautions that future studies should not have the blanket assumption of hyperexcitability in CA1 neurons across all AD models and at all ages, with each model and age requiring further examination.

## Data availability statement

The datasets presented in this study can be found in online repositories. The names of the repository/repositories and accession number(s) can be found below: https://github.com/sohline/aged-APP_PS1-CA1-excitability.

## Ethics statement

The animal study was reviewed and approved by the Animal Ethics Committee, University of Otago (AUP #20-91).

## Author contributions

SO wrote the first draft. SO, MI, and XL designed and performed the experiments and primary analysis. BM and WA provided the mice for the breeding colony. SO, WA, RE, KI, and PJ contributed to secondary analysis and obtained funding for the experiments. All authors edited drafts of the manuscript.

## Supplementary material

Supplementary Figure 1 Sample data used to determine input resistance (cf. Figure 2A). **(A)** The resulting voltage response to **(B)** seven current steps, from −100 pA to + 50 pA. Currents steps with resulting action potentials (i.e., +75 pA in this example) were not included. **(C,D)** Sample data for two WT cells, **(E,F)** sample data for two Tg cells. The resulting slope (input resistance) is shown above the line.
