# Supplementary figures and images for: Altered membrane properties but unchanged intrinsic excitability and spontaneous postsynaptic currents in an aged APPswe/PS1dE9 model of Alzheimer’s disease
Source: Front Cell Neurosci. 2022 Aug 26;16:958876. doi: 10.3389/fncel.2022.958876 (PMC9459330; doi:10.3389/fncel.2022.958876)

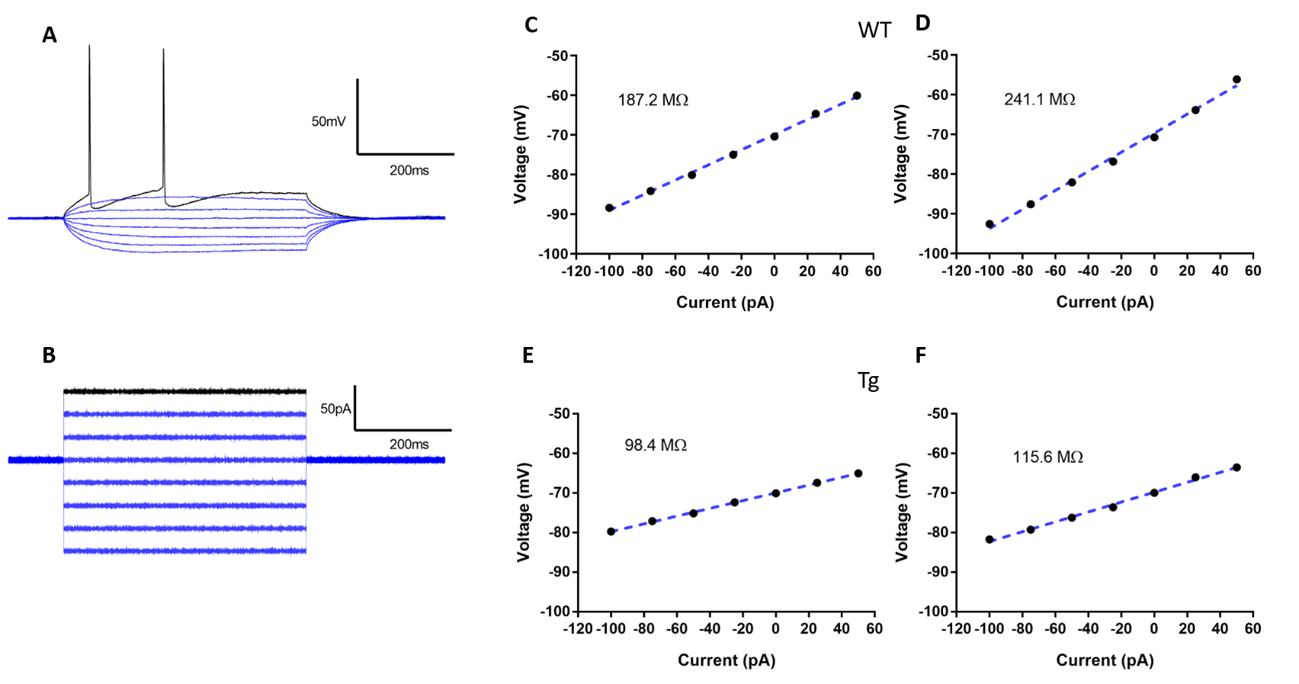

Supplement: Supplementary Figure 1 — Sample data used to determine input resistance (cf. Figure 2A). (A) The resulting voltage response to (B) seven current steps, from −100 pA to + 50 pA. Currents steps with resulting action potentials (i.e., +75 pA in this example) were not included. (C,D) Sample data for two WT cells, (E,F) sample data for two Tg cells. The resulting slope (input resistance) is shown above the line. [file Image_1.tif]
